# Impact of the recreational use of virtual reality on physical and mental wellbeing during the Covid-19 lockdown

**DOI:** 10.1007/s12553-021-00528-8

**Published:** 2021-02-14

**Authors:** Alessandro Siani, Sarah Anne Marley

**Affiliations:** School of Biological Sciences, University of Portsmouth, King Henry Building, King Henry 1st Street, PO1 2DY Portsmouth, UK

**Keywords:** Virtual reality, VR, Covid-19, Coronavirus, Lockdown, Mental health, Fitness

## Abstract

The Covid-19 pandemic has brought about significant changes to most aspects of our lives. As a result of the quarantine enforced by governments and authorities worldwide, people had to suddenly adapt their daily routines, including work, study, diet, leisure and fitness activities to the new circumstances. A growing body of research indicates that the engagement with virtual reality (VR) activities can have a positive impact on users’ mental and physical wellbeing. This study aims to evaluate the impact of VR activities on users under lockdown due to the Covid-19 pandemic. An online survey was carried out to investigate the recreational use of VR during the lockdown period and to gather users’ opinions on its impact on their physical and mental health. Non-parametric tests were used to evaluate the statistical significance of the responses provided by the 646 participants. The results of the survey show that VR use has significantly increased during the lockdown period for most participants, who expressed overwhelmingly positive opinions on the impact of VR activities on their mental and physical wellbeing. Strikingly, self-reported intensity of physical activity was considerably more strenuous in VR users than in console users. Given the current uncertainty as to the duration and course of the pandemic, as well as the possibility of intermittent lockdown in the upcoming years, the outcomes of this study could have a significant impact towards the development and deployment of VR-based strategies aimed at helping the population cope with prolonged social distancing, with particular regards to vulnerable individuals.

## Introduction

With over 52 million cases and 1.29 million confirmed fatalities so far, the ongoing Covid-19 pandemic has profoundly impacted all aspects of our lives. From forced lockdowns to social distancing and travel restrictions, the pandemic can be considered a watershed moment in human history. Aside from the obvious repercussions on those who contracted the disease and their loved ones, the SARS-CoV-2 virus has changed our daily personal and professional lives in an unprecedented manner; several studies have already reported significant impacts on the mental and physical health of the global population.

### Covid-19 and mental health

As recognised by the World Health Organization (WHO), the current outbreak poses a significant threat to global mental health [1]. Unsurprisingly, Covid-19 patients and Healthcare Workers (HCW) have been identified as the categories with the highest risk of suffering mental health consequences directly caused by the pandemic [2]. A large-scale longitudinal study showed a significant increase in mental distress amongst the UK population compared to the previous year, especially amongst young adults, women, and people living with young children [3]. While mental distress was understandably higher amongst lower-income households, a greater deterioration was observed in people that were employed before the pandemic than those who were unemployed. While this observation might look counterintuitive at a cursory glance, it could be a reflection of the stress caused by furlough or redundancy for dependant workers, or by the loss of business and income for self-employed ones. A review of the evidence published since the start of the pandemic highlighted that “the psychological impact of quarantine is wide-ranging, substantial, and can be long lasting” not only among patients and HCW, but also the general population [4]. The Covid-19 pandemic and ensuing containment measures (lockdown, quarantine, self-isolation, social distancing) have resulted in negative outcomes on public mental health, including “stress, depression, irritability, insomnia, fear, confusion, anger, frustration, boredom, and stigma associated with quarantine, some of which persisted after the quarantine was lifted” [5].

### Covid-19 and physical fitness

Movement restrictions have been enforced by most governments worldwide in an attempt to control the spread of the SARS-CoV-2 virus. Examples include travel bans between and within countries, closure of commercial activities and public spaces, and prolonged domestic isolation. The closure of gyms, sport pitches and parks, combined with heavy restrictions to outdoor movement, have forced millions of people to suddenly adopt an unusually sedentary lifestyle. The forced physical inactivity, together with the documented onset of unhealthy coping mechanisms such as smoking, drinking and food craving, represent a serious cause of concern in terms of fitness and wellbeing of the affected population [6, 7]. Examples of adverse health outcomes caused by physical inactivity include (but are not limited to) weight gain, increased risk of cardiovascular diseases (CVD), muscle atrophy, bone mass loss, reduced aerobic capacity, and the onset of metabolic disorders which constitute in and of themselves risk factors for a plethora of other deleterious conditions [8]. According to WHO guidelines, children and adolescents should dedicate at least 60 min a day to moderate or vigorous physical activity, whereas adults should aim to do at least 150 min of moderate physical activity or 75 min of vigorous activity a week [9]. Following the onset of the Covid-19 pandemic, the WHO launched the #HealthyAtHome campaign, and similar initiatives have been promoted by governments and national health services to provide advice and guidance on how to maintain a healthy lifestyle during the lockdown [10].

### Virtual Reality

Virtual Reality (VR) is defined by the Cambridge Dictionary as “a set of images and sounds, produced by a computer, that seem to represent a place or a situation that a person can take part in” [11]. Thanks to their increasing ease of use and affordable prices, VR headsets have recently gone from being a niche gadget for technology enthusiasts to a mainstream consumer product [12]. A wide range of VR applications have been developed for purposes as different as entertainment, education, business, and healthcare. VR has been used to improve users’ mental health in a number of scenarios. Recent studies indicate that VR-based strategies have successfully been used for the management of anxiety, phobias, stress, eating disorders, substance abuse, panic disorder, post-traumatic stress disorder, schizophrenia, bipolar disorder, psychosis, depression, and autism [13–15]. The ability to move freely in a virtual environment has paved the road to the use of VR for “exergaming” and fitness. An increasing body of evidence show that VR-based interventions can improve physical fitness, muscle strength, balance, extremity function, and overall quality of life in patients undergoing physical rehabilitation [16, 17]. Similar observations have been reported with regards to the beneficial impact of VR exercise regimes on other at-risk categories such as the elderly and individual suffering from mental health disorders and cognitive impairment [18–20].

### Aims of the study

This study aimed to evaluate the effectiveness of VR as a physical and mental health aid for people observing social distancing due to the Covid-19 pandemic. Towards that purpose, an online survey was carried out with the primary objective to investigate the recreational use of VR and gather the participants’ opinion on its impact on their mental and physical wellbeing. The secondary objective of the study was to compare the perceived effectiveness of VR fitness activities with that of non-VR exergames (e.g. Nintendo Wii/Wii U, Xbox Kinect, PlayStation move).

## Methods

An online survey was created using Google Forms and participants were recruited via social media from 26/5/2020 to 7/6/2020 using a convenience sampling strategy. In addition to the authors’ personal Facebook and Twitter feeds, the survey link was posted on relevant Facebook groups (Virtual Reality Society, Oculus Virtual Reality) and Reddit channels (r/virtualreality, r/VRGaming, r/SteamVR, r/PSVR, r/OculusQuest, r/GearVR, r/Vive). These pages were selected on the base of their accessibility, high number of active users and their potential interest in the topic of the survey.

The survey was prefaced by a disclaimer (available in the supplementary materials) describing the aims and nature of the study. Participants were explicitly invited to ensure that their answers reflected their situation during the Covid-19 lockdown, even if the lockdown had since been lifted. The questionnaire (Table 1) was composed of 16 questions, including closed (Likert-type, yes/no, multiple choice) and open questions. Ethical approval (reference number SFEC 2020–038) was obtained from the University of Portsmouth Faculty of Science & Health Ethical Committee prior to the start of the investigation. The survey was entirely anonymous and no information allowing the identification of individual participants was collected. Participants were informed of the voluntary and anonymous nature of the survey, as well as of their right to withdraw from it before submission. Data was collected, handled and stored in accordance with the General Data Protection Regulation.

**Table 1:** questionnaire used in the study. For the sake of conciseness, the options provided in multiple-choice questions are not shown in this table; they can however be found in the supplementary materials

Section 1: Participant information
1.1 What country do you live in?
1.2 What is your age range?
1.3 What is your gender?
1.4 Have you been observing social distancing due to the Covid-19 pandemic?
1.5 How has your body weight changed since the start of the lockdown?
1.6 Which are your favourite pastime activities? Tick all that apply
1.7 Do you own, or have access to, a Virtual Reality (VR) headset?
Section 2: Use of VR (restricted to participants who answered YES to question 1.7)
2.1 Which of the following Virtual Reality (VR) headsets do you own or have access to? Tick all that apply
2.2 What is your favourite VR app/activity during the Covid-19 quarantine?
2.3 How much do you agree with the following statements related to your use of VR during the Covid-19 quarantine? My use of VR has increased during the quarantine. VR helps me keeping myself occupied. VR activities are a good alternative to gym or outdoor fitness to keep fit during the lockdown. VR activities have a positive impact on my mental health.
2.4 Would you recommend VR as a pastime over other activities (TV, books, music, etc.)? Why or why not?
2.5 On average how much time a day do you spend using VR for the following activities?
2.6 When using VR for fitness activities, how would you rate the intensity of the activity?
Section 3: Console-based exergaming
3.1 Do you own, or have access to, a gaming console?
3.2 If YES, which of the following gaming consoles do you own or have access to? Tick all that apply
3.3 When using a gaming console for fitness activities (for example using Xbox Kinect, PlayStation Move, Nintendo Wii, etc.), how would you rate the intensity of the activity?

The Chi-square test is a non-parametric test suitable for the analysis of nominal data. As such, this was used to examine the proportion of respondents who selected different responses with regard to explanatory variables. All tests were conducted in R (version 4.0.2) with a significance cut-off value of 0.05. Due to the small number of responses from older age groups, age data were grouped into 18–19, 20–29, 30–39, 40 + categories.

## Results

### Study population

A total of 646 participants from 47 countries took part in the survey. The majority of participants were from the USA (n = 255), UK (n = 153), Canada (n = 51), Germany (n = 20), Australia (n = 17), the Netherlands (n = 13), Italy (n = 12) and Austria (n = 11). All other countries had less than 10 respondents each. A total of 641 respondents reported their gender, of which the majority were male (n = 535) or female (n = 94), and 12 identifying as ‘other’ (e.g. non-binary, transgender, gender fluid, etc.). Finally, 632 respondents indicated their age: 18–19 yo (n = 78), 20–24 yo (n = 91), 25–29 yo (n = 110), 30–34 yo (n = 126), 35–39 yo (n = 100), and 40 + yo (n = 127).

### Device access and usage

There was a significant association between VR access and gender (χ^2^ = 84.765, df = 2, p < 0.001), with 91.6% of males and 91.7% of others having access to a VR device in comparison to only 56.4% of females. However, there was no significant association between VR access and age, nor console access and age or gender (p > 0.05).

**Fig. 1:**
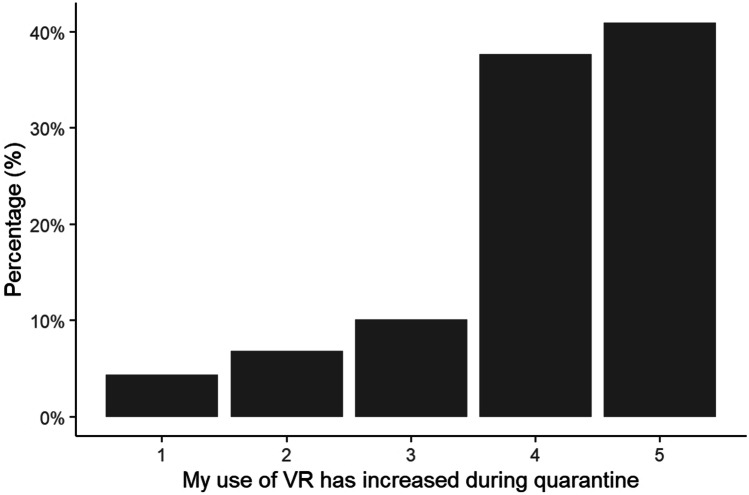
Percentage of respondents reporting whether their use of VR has increased during the Covid-19 lockdown on a scale from 1 (Strongly Disagree) to 5 (Strongly Agree)

**Fig. 2:**
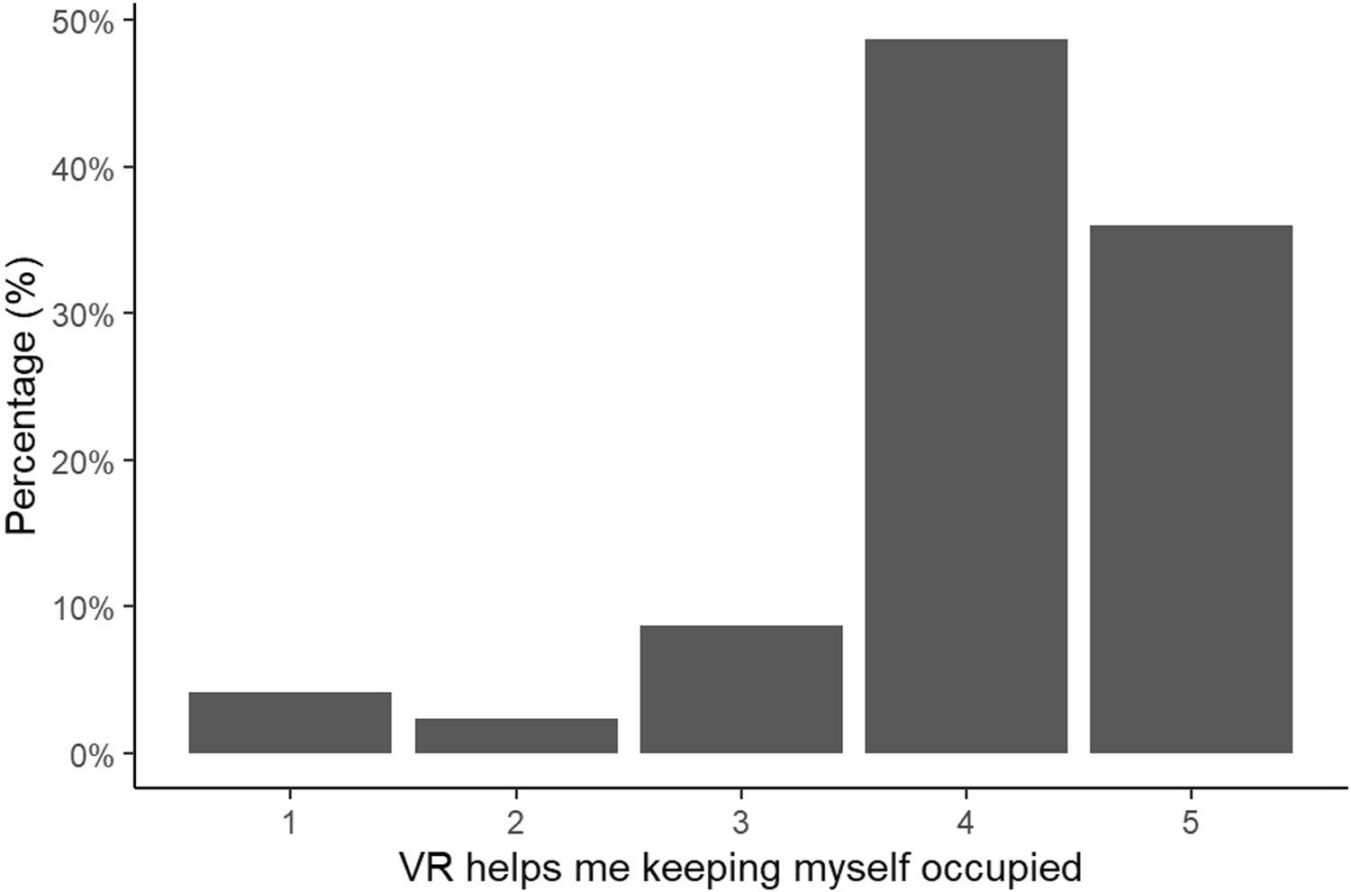
Percentage of respondents reporting whether VR has helped to keep themselves occupied during the Covid-19 lockdown on a scale from 1 (Strongly Disagree) to 5 (Strongly Agree)

Among respondents with VR access, the majority felt their usage of VR had increased during lockdown (χ^2^ = 351.83, df = 4, p < 0.001; Fig. 1) and that it helped to keep them occupied (χ^2^ = 489.34, df = 4, p < 0.001; Fig. 2). This was true for all gender and age categories (p > 0.05).

**Fig. 3:**
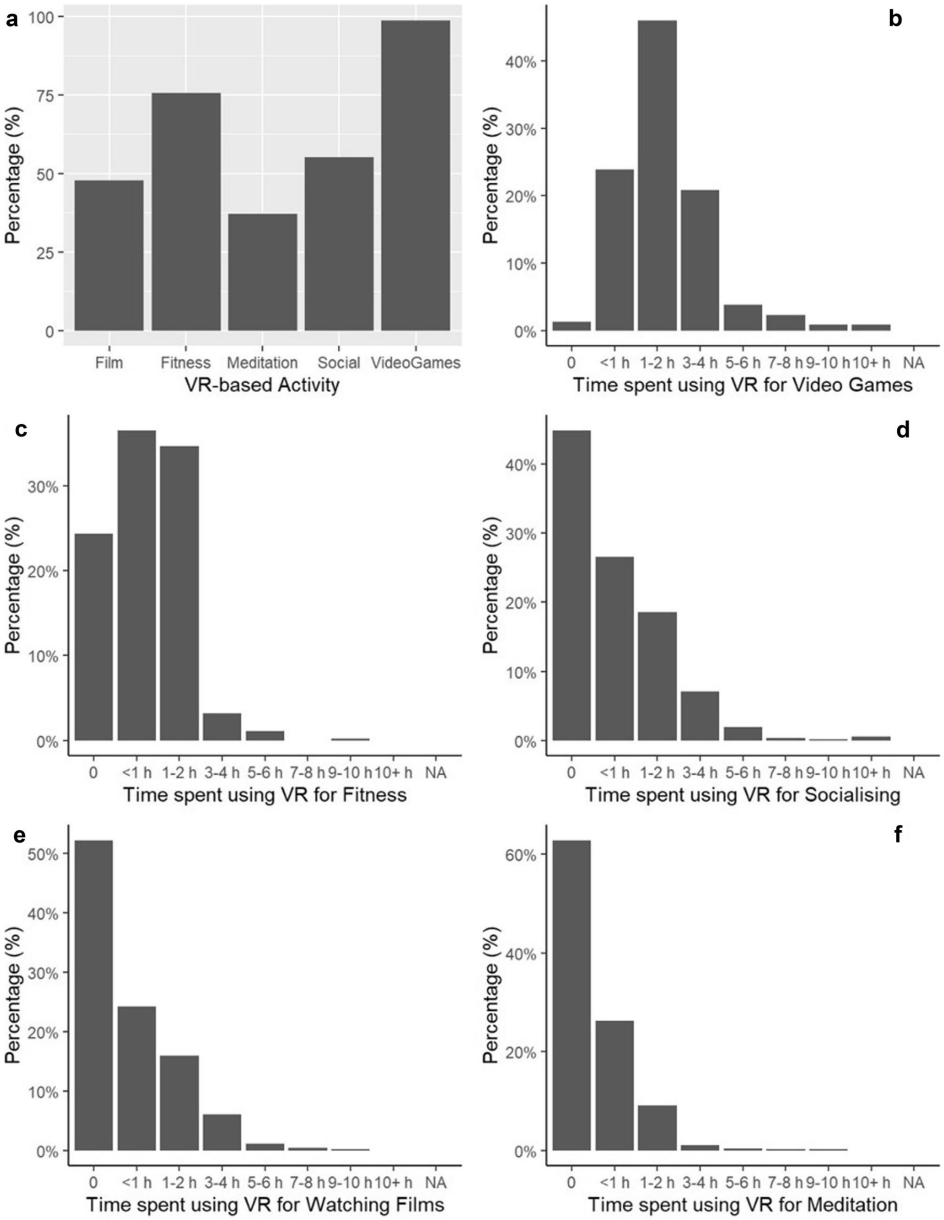
Percentage of respondents engaging in VR activities (a) and the proportion of time spent on those activities during the Covid-19 lockdown (b-f)

There was a significant difference in the proportion of respondents using VR for different activities (χ^2^ = 237.531, df = 4, p < 0.001; Fig. 3a). The most popular use of VR was for video games, with 98.7% of respondents using VR for this activity. This was followed by fitness (75.7%), socialising (55.2%), watching films (47.8%) and meditation (37.2%). Within activities, there were significant differences in how many hours per day people engaged (all p < 0.001; Fig. 3b-f). Of those people who spent time on each activity, the majority spent less than 1 h / day on fitness (48.2%), socialising (48.1%), watching films (50.6%) and meditation (70.7%). The exception was playing video games, where the majority (46.6%) of users spent 1 – 2 h / day on this activity.

**Fig. 4:**
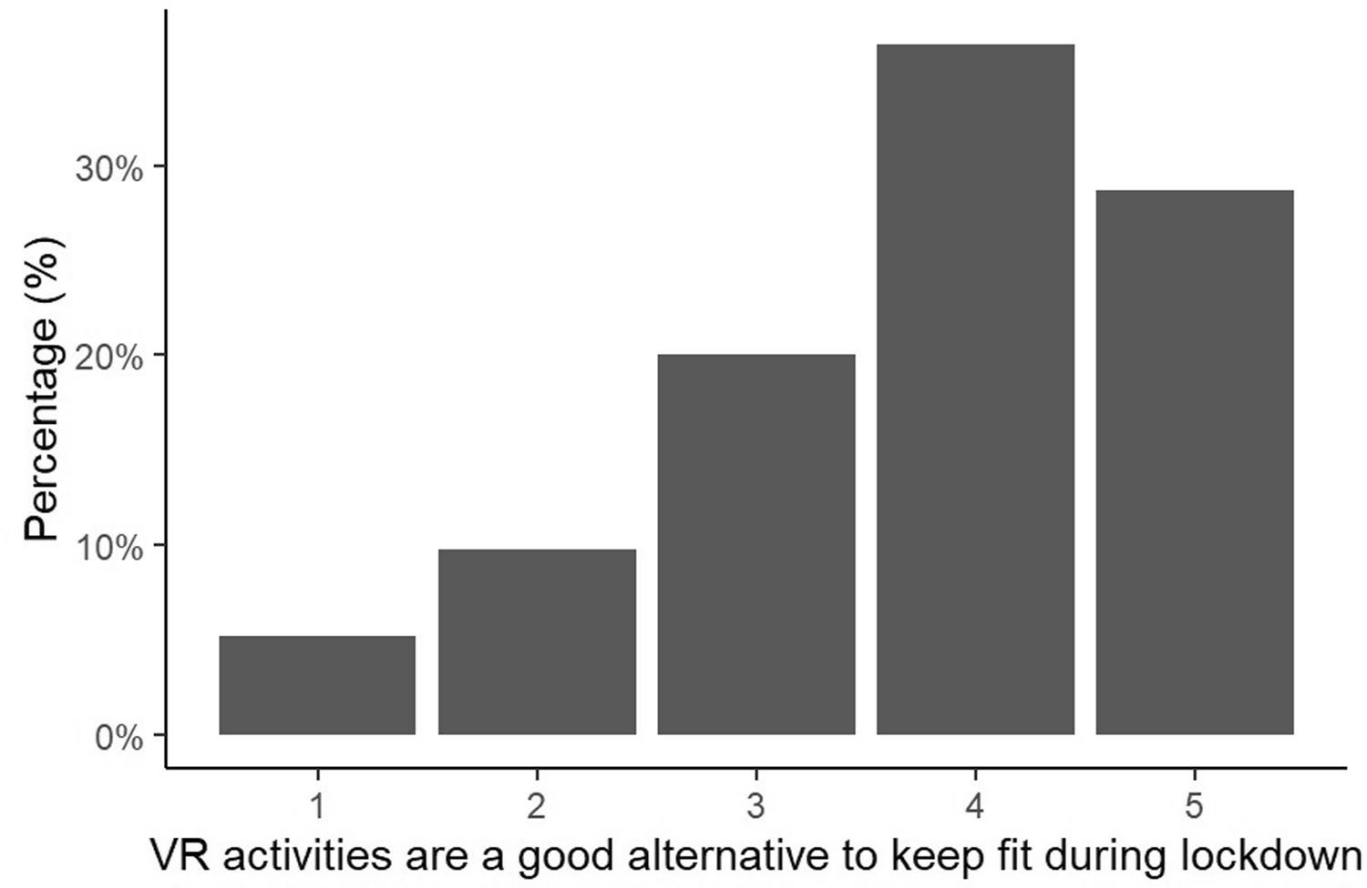
Percentage of respondents reporting that VR activities are a good way to keep fit during the Covid-19 lockdown on a scale from 1 (Strongly Disagree) to 5 (Strongly Agree)

Using VR to play video games was very popular with the majority of respondents (Fig. 3b). There was a significant difference for the amount of time spent playing video games according to gender (χ^2^ = 53.218, df = 14, p < 0.001) and age (χ^2^ = 88.707, df = 35, p < 0.001). The majority of females (43.1%) spent < 1 h / day playing video games, compared to the majority of males (47.4%) spending 1–2 h / day and the majority of others (36.4%) spending 3–4 h / day. With regards to age, the majority of 18–19 yo (36.5%), 20-24yo (38.7%), 25–29 yo (52.7%), 30–34 yo (41.5%), 35–39 yo (49.4%) and 40 + yo (53.2%) play between 1 – 2 h / day. The next highest proportion of respondents play video games between 3 – 4 h / day for 18–19 yo (29.7%) and 20–24 yo (32.0%); however, for the remaining age groups this drops to < 1 h / day (25–29 yo, 22.0%; 30–34 yo, 36.8%; 35–39 yo, 24.1%; 40 + yo, 24.8%).

There was also a significant difference in time spent socialising in VR according to gender (χ^2^ = 69.553, df = 14, p < 0.001) and age (χ^2^ = 55.127, df = 35, p < 0.001). The majority of ‘other’ respondents (36.4%) spent 1 – 2 h / day using VR for socialising, whilst females and males spent no time (42.9% and 45.6%, respectively) or very little (< 1 h / day; 28.6% and 26.5%, respectively). Using VR for socialising was most popular with 18-19yo (72.1%) and least popular with 25-29yo (47.7%).

There was a significant difference for time spent in VR for meditation activities according to age (χ^2^ = 44.157, df = 30, p < 0.001). Meditation was most popular with 40 + yo (46.8%) and least popular with 18–19 yo (24.2%). Where respondents were using VR for meditation activities, this was typically for < 1 h / day for all age groups.

There were no gender-related differences for meditation activities, nor gender- or age-related differences in the time spent on VR for fitness or watching films (all p > 0.05).

### Wellbeing

**Fig. 5:**
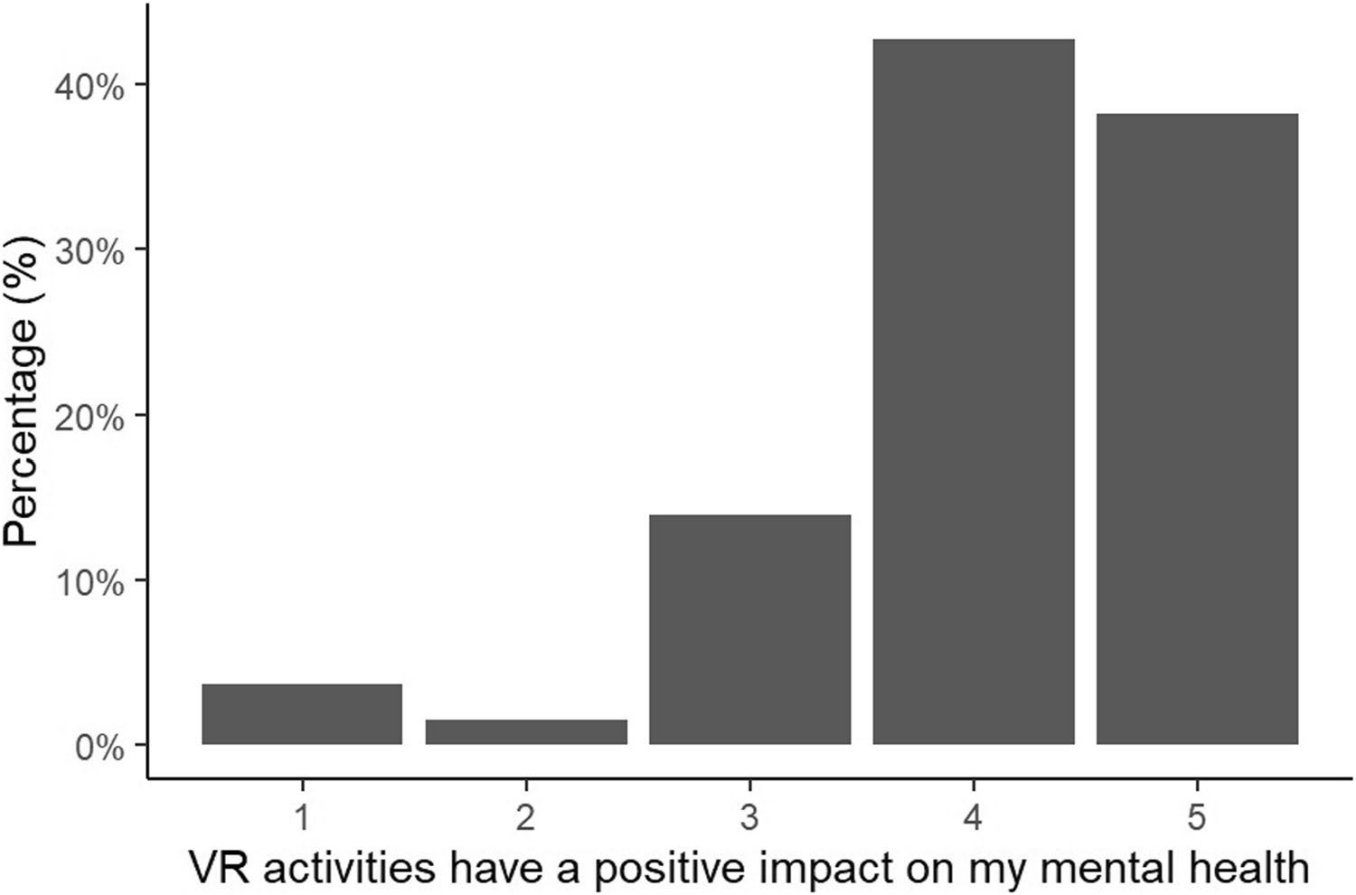
Percentage of respondents reporting that VR activities have a positive impact on their mental health on a scale from 1 (Strongly Disagree) to 5 (Strongly Agree)

Of those respondents with VR Access, the majority were positive about the usefulness of VR for fitness (χ^2^ = 185.21, df = 4, p < 0.001; Fig. 4) and mental health (χ^2^ = 416.27, df = 4, p < 0.001; Fig. 5). This was true for all gender and age categories (p > 0.05).

**Fig. 6:**
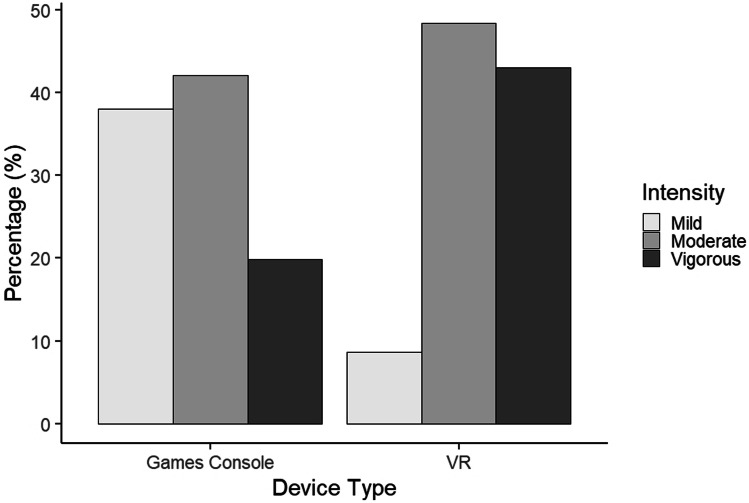
Self-reported fitness intensity by device type. Traditional gaming consoles (e.g. Xbox, PlayStation, Nintendo) are shown on the left, VR headsets on the right

### Intensity of fitness activities

There was a significant difference in self-reported intensity of physical activities according to device type (χ^2^ = 102.256, df = 2, p < 0.001; Fig. 6). The majority of both VR (48.4%) and console (42.1%) users engaged with moderate intensity. However, a greater proportion of VR users engage in vigorous activity (43.0%) than mild activity (8.6%), a trend which is reversed in console users (38.0% mild, 19.8% vigorous).

### Body Weight

**Fig. 7:**
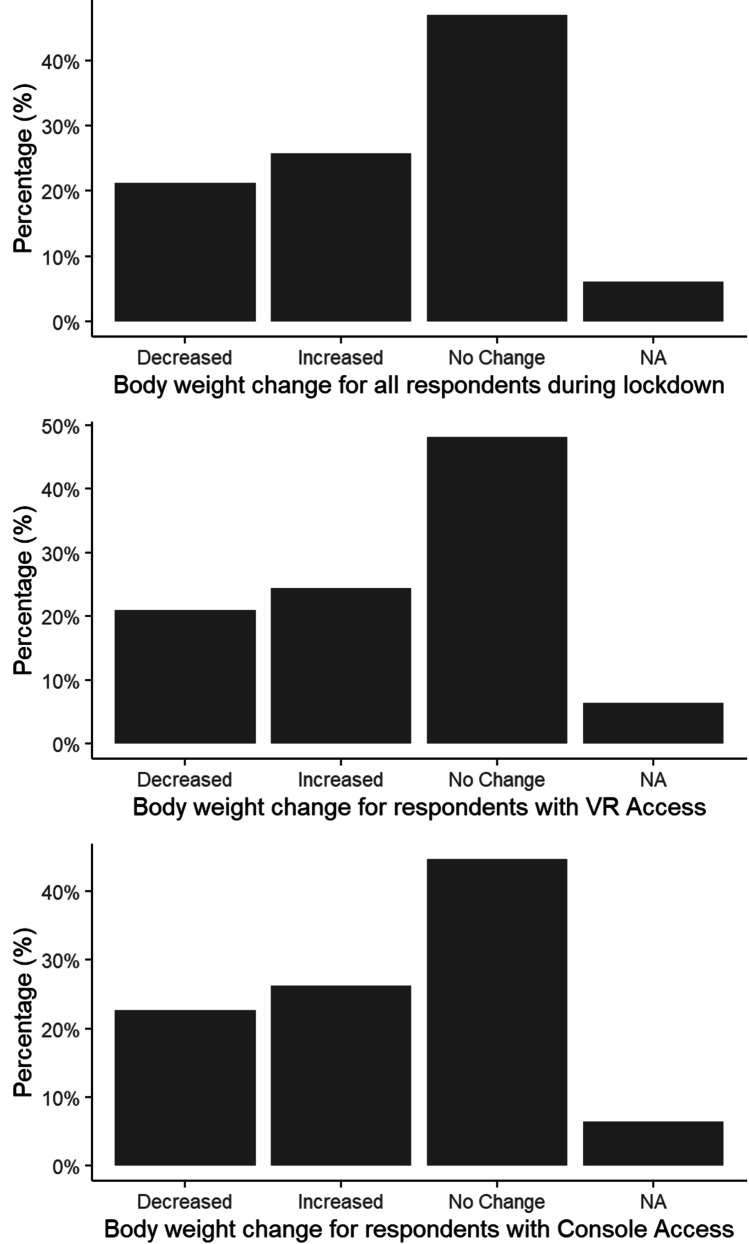
Self-reported weight change for all participants (top), for respondents with VR Access (middle) and for those with console access (bottom

A significant difference was observed in the proportion of participants reporting weight gain, weight loss, or no weight change during lockdown (χ^2^ = 76.962, df = 2, p < 0.001; Fig. 7, top). Almost half (49.9%) of respondents did not experience any change in body weight, whilst 22.6% experienced weight loss and 27.4% experienced weight gain.

Of those respondents with VR Access, the majority reported no change (51.4%) or a decrease (22.5%) in their weight (χ^2^ = 77.900, df = 2, p < 0.001; Fig. 7, middle). For these respondents there was no association between body weight and VR Fitness Time or VR Fitness Intensity (p > 0.05).

Similarly, of those respondents with console access, the majority also showed no change (47.7%) or a decrease (24.2%) in their weight (χ^2^ = 47.403, df = 2, p < 0.001; Fig. 7, bottom). But again, there was no association between body weight change and console fitness Intensity (p > 0.05).

## Discussion

The results of this study support the hypothesis that the recreational use of VR can have a positive impact on mental and physical wellbeing during periods of forced lockdown. The vast majority of participants, regardless of their age and gender, reported that their use of VR has increased during lockdown, and agreed that this has helped keeping themselves occupied.

Amongst the participants of the study, video games were the most popular VR activity, followed by fitness, social media, videos, and meditation. Nearly 70% of the participants reported spending 1 to 4 h a day playing video games in VR and a similar percentage reported using VR for fitness purposes for up to 2 h a day. It is worth pointing out that, as many VR video games involve a significant physical component, there might be some overlap between the two categories, i.e. it is likely that a large part of the time spent exercising refers to users doing so while gaming. A lower percentage of participants (< 20% in all cases) reported spending more than one hour a day using VR for video, social, or meditation activities. These observations are consistent with recent findings that regularly engaging even with short VR sessions (e.g. a 10-min 3D 360-degree video every day for a week) can alleviate lockdown-induced anxiety and foster a positive mindset [21].

The majority of the population surveyed in this study, regardless of their gender and age, reported that their VR use has increased during the lockdown, and expressed overwhelmingly positive opinions on the usefulness of VR as a way to keep busy and improve their mental and physical wellbeing. While previous studies had reported that VR activities can be effective in improving psychophysical wellbeing in older adults [22], our study is, to the best of our knowledge, the first to investigate its impact on adults across all age groups with specific regards to the lockdown environment.

A striking difference was observed in self-reported exercise intensity between participants using gaming consoles and VR headsets, with the latter being associated to considerably more intense physical activity compared to the former. Among console users, 38% defined their exercise intensity as “mild”, and 19.8% as “vigorous”. On the other hand, only 8.6% of VR users described their exercise intensity as “mild”, whereas 43% of them engaged with vigorous exercise. These results indicate that VR may be a more effective “exergaming” device than traditional consoles. Similarly to the case discussed in the previous paragraph, while the effectiveness of exercising via VR and gaming consoles in older adults had been previously investigated in peer-reviewed literature [23], the present study is the first to evaluate and compare the two systems across all adult age groups.

Regardless of their age, gender, and VR/console use, the majority of participants reported no changes in body weight over the lockdown period. At a cursory glance, this observation may seem to conflict with the fact that many participants reported to regularly use VR for fitness purposes and described their exercise as moderate or vigorous. However, it is worth stressing that body weight change is underpinned by a multitude of factors such as nutrition, metabolic activity, and body composition in terms of fat and muscle mass [24]. As a result, while self-reported exercise intensity and weight changes are considered useful indicators of the overall fitness of a person [25], further elements (e.g. food diary, metabolic blood tests, bioelectrical impedance analysis, etc.) could be considered to obtain a more accurate and holistic picture.

## Conclusions

This study provides novel insight on how the recreational use of VR can successfully alleviate the negative impact of lockdown periods on the population’s mental and physical wellbeing. The results of the survey indicate that VR activities help users keep themselves occupied and physical active under the restrictions imposed by the lockdown. VR headsets have now become mainstream entertainment devices in many households due to their increasingly affordable price and technological accessibility [12]. Therefore, their potential as public health aids should be considered by researchers, policymakers, and healthcare workers to design and implement intervention strategies aimed at mitigating the negative consequences of prolonged lockdown periods. Providing the population with the means to engage with VR activities to keep them occupied and physically fit could be a promising strategy to minimise the decline in mental and physical wellbeing that has been reported in many instances since the start of the pandemic [26]. This type of self-administered intervention could potentially ease the current strain on healthcare and social support workers [27]. However, a cost/impact analysis would be required to assess the feasibility and effectiveness of replacing or supplementing part of the current in-person support strategies with ad-hoc VR-based programmes. While this study provides novel and significant evidence supporting the usefulness of VR activities to improve the population’s psychophysical health, it is limited by the fully self-reported nature of the survey, which is known to carry inherent risks of response bias [28]. Further studies would be advisable to monitor key physiological and psychological indicators of physical and mental wellbeing under controlled conditions.

## Availability of data and material

The dataset analysed in this study is available from the corresponding author on reasonable request.

## Supplementary Information

Below is the link to the electronic supplementary material.

Supplementary file1 (DOCX 19 KB)
