# Supplementary material for: Impact of the recreational use of virtual reality on physical and mental wellbeing during the Covid-19 lockdown
Source: Health Technol (Berl). 2021 Feb 14;11(2):425–35. doi: 10.1007/s12553-021-00528-8 (PMC7882463; doi:10.1007/s12553-021-00528-8)
Supplement: Supplementary file 1 — Supplementary file1 (DOCX 19 KB) [file 12553_2021_528_MOESM1_ESM.docx]

# Supplementary Information

Health and Technology

Impact of the recreational use of Virtual Reality on physical and mental wellbeing during the Covid-19 lockdown

# Survey preface

Dear Sir/Madam,

As a part of my research at the [REDACTED TO ANONYMISE], I am investigating the impact of Virtual Reality (VR) and gaming consoles on the mental and physical wellbeing during the Covid-19 lockdown.

I would like to ask you to kindly contribute to this study by filling this anonymous online survey that should take less than 5 minutes to complete. Your responses will be kept confidential and no identifying information such as your name, e-mail address or IP address will be collected. To participate, you need to be at least 18 old and live in a country where a lockdown has been enforced in response to the Covid-19 pandemic.

Your participation in this research study is entirely voluntary. You may choose not to participate. If you decide to participate in this research survey, you may withdraw at any time by just leaving the survey page before clicking the “submit” button. The answers will not be recorded unless you click "submit" at the end of the questionnaire, so make sure to do so if you wish to participate. By clicking the “submit” button, you consent to the use of your anonymous answers for the scopes of this research.

If you have any questions, suggestions or complaints about this study, do not hesitate to contact me at [REDACTED TO ANONYMISE].

Your help in this matter is greatly appreciated!

[REDACTED TO ANONYMISE]

# Note to participants

This questionnaire is designed to capture a snapshot of life during the Covid-19 lockdown period. As a result, please ensure that your answers reflect your situation during the lockdown period, even if the lockdown has now been lifted.

# Questionnaire

Section 1: Participant information

- 1. What country do you live in?

(a list of all countries was provided for participants to choose from)

1.2 What is your age range?

- 18-19
- 20-24
- 25-29
- 30-34
- 35-39
- 40-44
- 45-49
- 50-54
- 55-59
- 60-64
- 65-69
- 70-74
- 75-79
- 80-84
- 85-89
- 90 or more
- Prefer not to answer

1.3 What is your gender?

- Female
- Male
- Nonbinary
- Other (please specify)
- Prefer not to answer

1.4 Have you been observing social distancing due to the Covid-19 pandemic?

- Yes
- No
- Prefer not to answer

1.5 How has your body weight changed since the start of the lockdown?

- My body weight has increased
- My body weight has decreased
- My body weight has not changed
- Don't know/prefer not to answer

1.6 Which are your favourite pastime activities? Tick all that apply.

- Films/TV series/documentaries/other videos
- Games
- Meditation/Relax
- Fitness
- Social
- Crafting/cooking
- Reading
- Other (please specify)

1.7 Do you own, or have access to, a Virtual Reality (VR) headset?

- Yes
- No

Section 2: Use of VR (restricted to participants who answered YES to Question 1.7)

2.1 Which of the following Virtual Reality (VR) headsets do you own or have access to? Tick all that apply.

- Google Cardboard (or other 'cardboard' devices)
- Oculus Go/Quest/Rift
- Samsung Gear VR
- HTC Vive/Pro/Cosmos
- PlayStation VR
- Valve Index
- Other (please specify)

2.2 What is your favourite VR app/activity during the Covid-19 quarantine?

2.3 How much do you agree with the following statements related to your use of VR during the Covid-19 quarantine?

- My use of VR has increased during the quarantine.
- VR helps me keeping myself occupied.
- VR activities are a good alternative to gym or outdoor fitness to keep fit during the lockdown.
- VR activities have a positive impact on my mental health.

2.4 Would you recommend VR as a pastime over other activities (TV, books, music, etc.)? Why or why not?

2.5 On average how much time a day do you spend using VR for the following activities

Films/TV series/documentaries/other videos.

- Video games.
- Meditation/Relax
- Fitness
- Social

2.6 When using VR for fitness activities, how would you rate the intensity of the activity?

- Mild (activities that require physical effort, comparable to walking slowly or doing light housework)
- Moderate (activities that require moderate physical effort and cause small increases in breathing or heart rate, comparable to walking briskly, slow dancing, or vacuuming the floor)
- Vigorous (activities that require hard physical effort and cause large increases in breathing or heart rate, comparable to running, swimming, or carrying heavy loads)

Section 3: Console-based exergaming

3.1 Do you own, or have access to, a gaming console?

- Yes
- No

3.2 If YES, which of the following gaming consoles do you own or have access to? Tick all that apply.

- Sony PlayStation (1 to 4)
- Microsoft XBox (XBox, XBox 360, XBox one)
- Nintendo Wii/Wii U
- Nintendo Switch
- Other (please specify)

3.4 When using a gaming console for fitness activities (for example using Xbox Kinect, PlayStation Move, Nintendo Wii, etc.), how would you rate the intensity of the activity?

- Mild (activities that require physical effort, comparable to walking slowly or doing light housework)
- Moderate (activities that require moderate physical effort and cause small increases in breathing or heart rate, comparable to walking briskly, slow dancing, or vacuuming the floor)
- Vigorous (activities that require hard physical effort and cause large increases in breathing or heart rate, comparable to running, swimming, or carrying heavy loads)
